# Enhancing Paraoxon Binding to Organophosphorus Hydrolase Active Site

**DOI:** 10.3390/ijms222312624

**Published:** 2021-11-23

**Authors:** Léa El Khoury, David L. Mobley, Dongmei Ye, Susan B. Rempe

**Affiliations:** 1 Department of Pharmaceutical Sciences, University of California, Irvine, CA 92697, USA; lea_elkhoury@hotmail.com (L.E.K.); dmobley@uci.edu (D.L.M.); 2 Department of Chemistry, University of California, Irvine, CA 92697, USA; 3 Sandia National Laboratories, Albuquerque, NM 87123, USA

**Keywords:** organophosphorus hydrolase, organophosphorus compounds, mutagenesis, molecular dynamics simulations, binding mode, kinetic assays

## Abstract

Organophosphorus hydrolase (OPH) is a metalloenzyme that can hydrolyze organophosphorus agents resulting in products that are generally of reduced toxicity. The best OPH substrate found to date is diethyl p-nitrophenyl phosphate (paraoxon). Most structural and kinetic studies assume that the binding orientation of paraoxon is identical to that of diethyl 4-methylbenzylphosphonate, which is the only substrate analog co-crystallized with OPH. In the current work, we used a combined docking and molecular dynamics (MD) approach to predict the likely binding mode of paraoxon. Then, we used the predicted binding mode to run MD simulations on the wild type (WT) OPH complexed with paraoxon, and OPH mutants complexed with paraoxon. Additionally, we identified three hot-spot residues (D253, H254, and I255) involved in the stability of the OPH active site. We then experimentally assayed single and double mutants involving these residues for paraoxon binding affinity. The binding free energy calculations and the experimental kinetics of the reactions between each OPH mutant and paraoxon show that mutated forms D253E, D253E-H254R, and D253E-I255G exhibit enhanced substrate binding affinity over WT OPH. Interestingly, our experimental results show that the substrate binding affinity of the double mutant D253E-H254R increased by 19-fold compared to WT OPH.

## 1. Introduction

Organophosphorus hydrolase (OPH) is a bacterial enzyme that can detoxify a wide range of organophosphorus (OP) agents by hydrolyzing various phosphorus-ester bonds (P-O, P-F, P-CN, and P-S). OP compounds are toxic molecules used primarily as pesticides and nerve agents. OP compounds cause severe neurotoxic effects by covalently binding to acetylcholinesterase (AChE), an enzyme that catalyzes the breakdown of neurotransmitters such as acetylcholine. Binding by OP inhibits AChE, resulting in accumulation of neurotransmitter and rapid death in insects and humans due to lost control of respiratory muscles [1]. Current medical countermeasures, including atropine and oxime-based reactivators [2] that target the down-stream pathways of OP, act through reactivation of AChE, blocking acetylcholine receptor activity or simply easing the symptoms. A direct method that can hydrolyze OP agents before they enter the central nervous system is needed. Currently, OPH is the only enzyme used in organophosphate remediation [3], but more investigation is needed to improve the use of OPH in medical therapy. The advantage of using OPH therapy over oxime-based treatment is rapid hydrolysis of OP agents in the circulatory system. Thus, nerve agents are eliminated before penetrating the blood-brain barrier and exerting effects in the nervous system.

While the hydrolytic products of OP breakdown by OPH are generally of reduced toxicity relative to their parent compounds [3,4], substrate binding affinity is inadequate for therapy. Many traditional protein engineering studies have shown that inducing mutations in or near the active site residues can increase the catalytic efficiency (*k*_cat_/*K*_M_) by increasing the catalytic rate constant (*k*_cat_) of the enzyme [5]. Those efforts, however, resulted in OPH variants with reduced effectiveness for hydrolysis of multiple nerve agents. In particular, the wild-type OPH and variants currently available demonstrate poor substrate binding affinity (*K*_M_). Poor binding affinity means that the substrate concentration required for enzymatic conversion exceeds the nerve agent lethal dose. Therefore, an important topic of research is understanding how the structure of OPH correlates with substrate binding.

One hypothesis aimed at improving *K*_M_ is that stabilization of substrate binding in the OPH active site could increase the probability of transition state formation for OP conversion. To test this hypothesis, we augmented traditional protein approaches with computational approaches. Using molecular dynamics simulations, we analyzed the wild-type (WT) OPH active site. The analysis led to identification of amino acid residues that stabilize the OPH active site. Experiments tested the molecular dynamics predictions.

**Figure 1:**
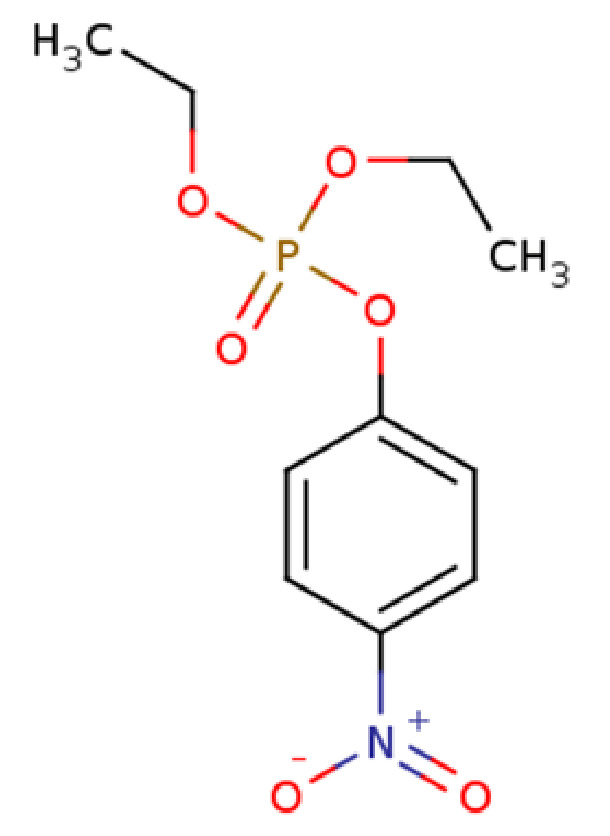
Representation of the 2D structure of paraoxon, which was generated using the ChemDB Chemoinformatics Portal (http://chemdb.ics.uci.edu), accessed on 11 November 2021.

A challenge in analyzing the OPH active site is that the native substrate remains unknown, and only structures with substrate analogs exist. While paraoxon (diethyl p-nitrophenyl phosphate) is considered a near-native substrate of OPH [6,7,8] (Figure 1), and induces neurotoxicity through the same manner as nerve agents by inhibiting AChE, it is also safe to test in research labs, which is an important consideration. It is not possible to obtain a co-crystal structure of the WT-OPH with the bound paraoxon because of the likelihood of hydrolysis. A co-crystal structure of OPH with diethyl 4-methylbenzylphosphonate (DMBP), which is a substrate analog sharing a high similarity with paraoxon, is available [9]. Consequently, most researchers use the binding mode of DMBP as a starting structure in their studies in order to model paraoxon [7,10,11]. In the absence of a bound structure, however, the validity of this analogy is uncertain. Thus, careful study of the paraoxon binding mode, and paraoxon’s potential interactions within the OPH binding site, is needed.

Before performing mutagenesis on OPH bound to paraoxon, we used docking and ligand-based methods, combined with molecular dynamics (MD) simulations, to model possible binding modes of paraoxon in the OPH binding site. Overall, we found a stable binding pose of paraoxon, which differs from the native binding mode of the substrate analog. Moreover, using the predicted binding mode, we performed MD simulations on different OPH mutants and found three different residues, D253, H254, and I255, involved in the stability of the catalytic site. Here, we report a detailed computational structural analysis on five mutant complexes that include those residues (D253E, D253E-H254R, D253E-I255G, H254S, and I255S).

Additionally, we tested the accuracy of our predictions using an experimental mutagenesis approach. When expressed experimentally, all designed single and double mutations (H254S, I255S, D253E, D253-H254R, and D253E-H254R) achieved improved substrate binding affinity. Our kinetics results showed, on the one hand, a substantial increase in substrate binding affinity of 19-, 10-, 5.2-, and 4.8- folds to D253E-H254R, D253E, H254S, D253E-I255G, respectively. The designed single mutation I255S, on the other hand, presented a modest increase of substrate binding affinity (1.6 fold) compared to the WT-OPH.

## 2. Results and Discussion

Despite several engineered variants, applications of OPH suffer from the enzyme’s low substrate binding efficiency, hindering its action as an OP scavenger. Here, we focused on designing OPH mutants that yield stronger substrate binding while minimizing the reduction of catalytic effectiveness. Many previous studies have been done to improve OPH substrate binding through rational design or direct evolution [5,12,13]. None of the resulting designed variants, which mainly targeted active-site modifications, improved binding properties to nerve agents sufficiently to prevent lethality.

**Figure 2:**
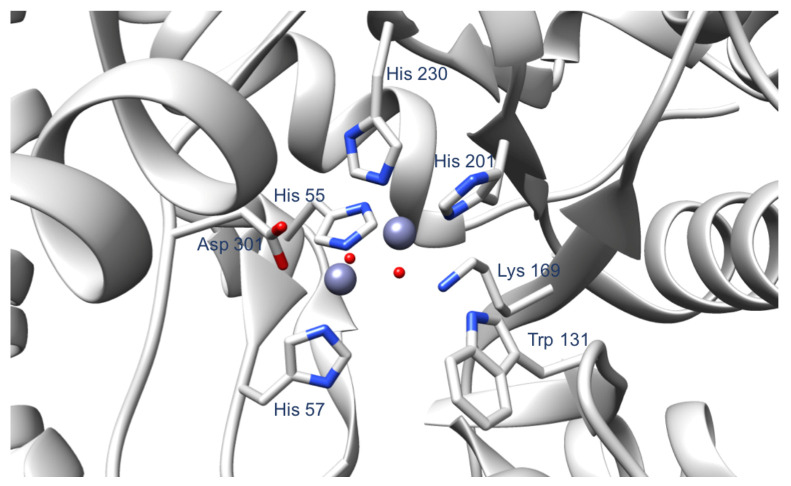
The OPH active site, highlighting the amino acid residues that coordinate the two Zn(II). Other important residues involved in the catalytic activity of the enzyme are Trp 131 and Lys 169. The PDB used to produce this figure is 1HZY. The Zn(II) ions and the water molecules are shown in purple and red spheres, respectively.

In the current work, we targeted residues surrounding the OPH active site (Figure 2). The selected residues do not coordinate the Zn(II) cations in the active site, and are not directly involved in the hydrolysis reaction. We hypothesized that the alterations of these residues would stabilize the OPH active site by increasing the hydrogen bonding associated with the side chains of the active site residues, thus leading to a more stable substrate binding. In contrast with previously designed OPH variants focused on modification of the active site residues to increase the catalytic activity of OPH, our current findings suggest that mutations near the enzyme active site can enhance paraoxon binding affinity.

### 2.1. Paraoxon Binding Mode

**Figure 3:**
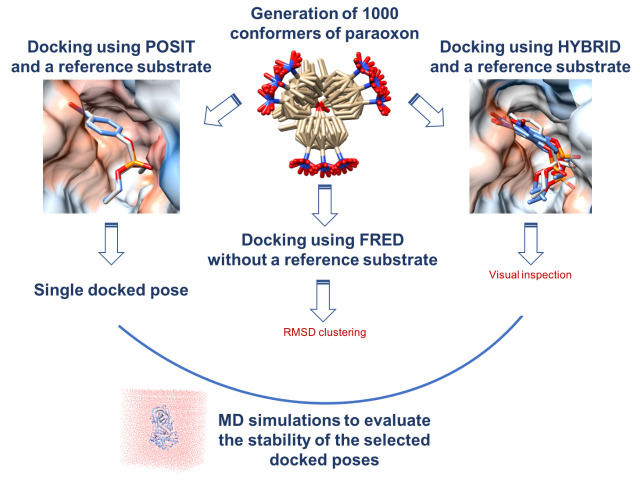
A schematic description of our docking protocol involving three different docking tools (POSIT, HYBRID, and FRED) from OpenEye Toolkits used to dock paraoxon in the active site of OPH.

Prior to mutagenesis of the OPH residues, we wanted to predict the correct binding mode of paraoxon in the OPH active site. As described in the Methods section, we performed docking using three pieces of docking software and selected several representative docking poses (Figure 3).

**Figure 4:**
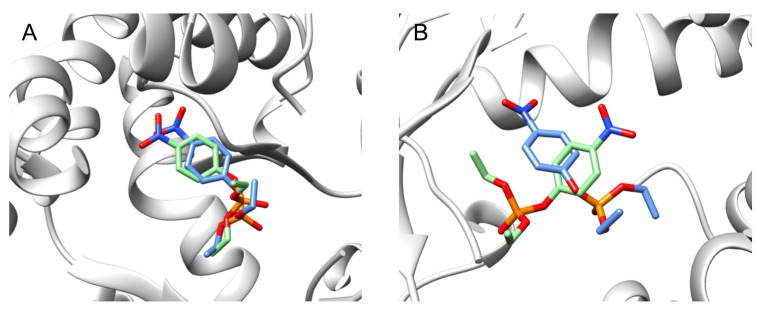
Superimposition of the docking poses before (in green) and after (in blue) MD simulations within the OPH catalytic site. To evaluate the stability of our docking poses during MD simulations, we calculated the RMSD of each pose at the end of the MD trajectories (15 ns) relative to the starting pose. Here, we report in: (**A**) the most stable binding pose with the lowest RMSD value of 1.77 Å; this pose was generated using HYBRID, and (**B**) the docking pose that reproduces the binding mode of the substrate analog; it was generated using POSIT (OEDocking 3.2.0; OpeneEye Inc.). Contrary to our expectations, we found that this binding mode is not stable (RMSD of 6.6 Å), highlighting that the binding mode of the substrate analog does not reflect the binding mode of paraoxon.

Then, we ran short MD simulations on the selected poses to assess their stability in the OPH active site. For this purpose, we calculated the RMSD of the poses at the end of the 15 ns long MD trajectories with respect to the starting poses before MD, and found that the pose with the lowest RMSD value (1.77 Å) was generated by HYBRID (Figure 4A). Interestingly, we found that the docking pose generated using POSIT, which best overlays the structure of diethyl 4-methylbenzylphosphonate, is not stable and scores an RMSD of 6.6 Å (Figure 4B). Essentially, while the ligand can *dock* in an overlapping pose, our simulations indicate it is not stable in that binding mode and that other binding modes are more favorable/stable. In contrast to previous assumptions, this finding suggests that the binding mode of paraoxon is different than that of 4-methylbenzylphosphonate.

### 2.2. The Single Mutant D253E

**Figure 5:**
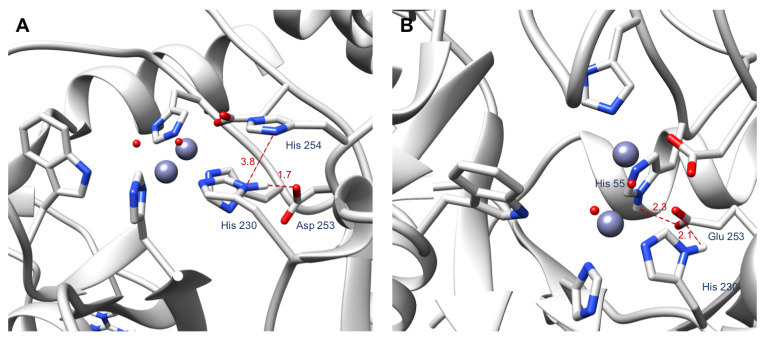
The binding site of OPH. (**A**) Figure showing the hydrogen bond between His 230 and Asp 253 and the stacking between His 230 and His 254. (**B**) Figure showing the hydrogen bonds that Glu 253 establishes with His 55 and His 230. The Zn(II) ions and the water molecules are represented in purple and red spheres, respectively. Only the hydrogen atoms involved in the highlighted interactions are shown.

In wild type OPH, the carboxylate group of Asp 253 forms a hydrogen bond with the imidazole of His 230 (Figure 5A). This hydrogen bond orients His 230 for an optimal coordination of one of the Zn(II) in the active site. We computationally mutated the aspartate residue at position 253 to a glutamate to create two hydrogen bonds with two residues in the active site (His 230 and His 55) (Figure 5B), instead of the single hydrogen bond in the WT OPH structure.

**Figure A1:**
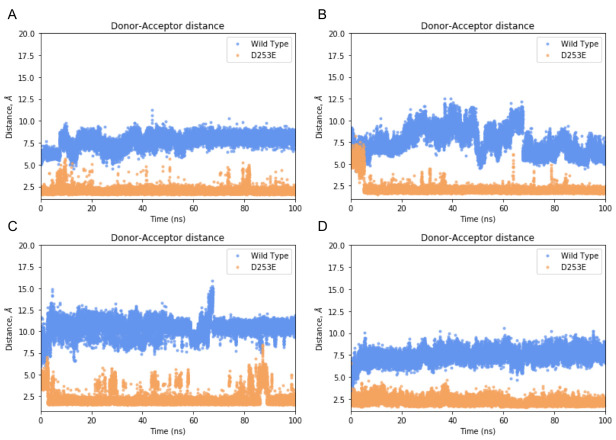
The donor-acceptor distances found during 100 ns of MD simulation in Wild Type OPH complexed to paraoxon and the mutant D253E complexed to paraxon between: (**A**) Thr 54 and His 55, (**B**) His 57 and Trp 302, (**C**) Thr 200 and His 201, and (**D**) Leu 271 and His 254. We found that, during the simulations on the OPH variant (D253E) complex, the distances between: (i) Thr 54 and His 55, (ii) His 57 and Trp 302, (iii) Thr 200 and His 201, and Leu 271 and His 254 are short (fluctuating around 2 Å), signaling the formation of hydrogen bonds between the residues belonging to each of these pairs. The respective donor-acceptor distances during MD simulation on the WT complex are greater than the typical distance of a hydrogen bond (2 Å). These results show that the mutation D253E induced four different hydrogen bonds near the active site of OPH. Pictorial representations of these interactions are shown on Figure A2.

**Figure A2:**
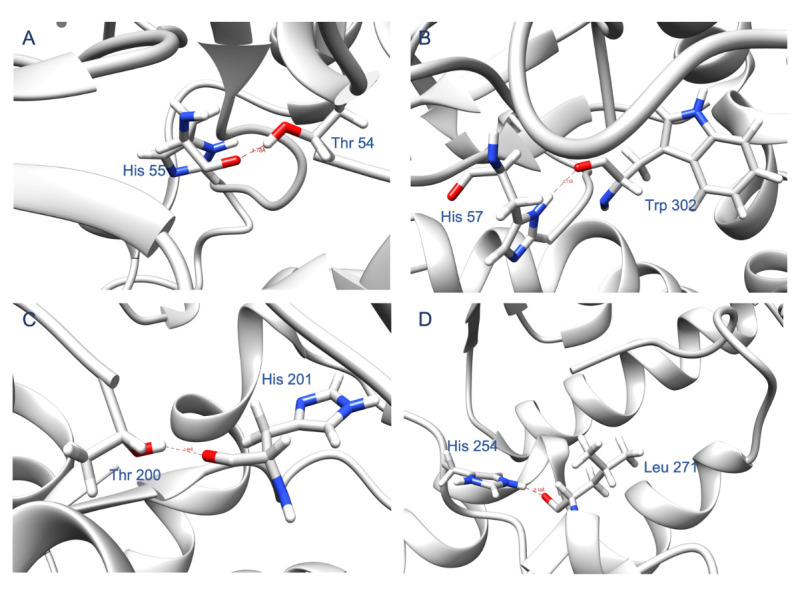
The donor-acceptor distances found during MD simulation of the mutant D253E complexed to paraxon between: (**A**) Thr 54 and His 55, (**B**) His 57 and Trp 302, (**C**) Thr 200 and His 201, and (**D**) Leu 271 and His 254. These results show that the mutation D253E induced four different hydrogen bonds near the active site of OPH.

Then, we performed MD simulations on the WT-OPH complexed with paraoxon and the D253E mutant complexed with paraoxon to evaluate whether the substitution of the aspartate’s side chain by a longer side chain would have an impact on the number and the stability of the interactions between the OPH active site residues. A structural analysis of our MD simulations showed that, in the mutant form D253E, the active site residues establish four additional stable hydrogen bonds compared to the WT OPH. As shown on Figure A1 and Figure A2, and based on the distance between atoms that could form potential hydrogen bonds during MD simulations, we concluded that four interactions are more stable during the simulations of the D253E mutant complex. These interactions occur between: (i) Thr 54 and His 55, (ii) His 57 and Trp 302, (iii) Thr 200 and His 201, and (iv) Leu 271 and His 254. His 55, His 57, and His 201 take part in the Zn(II) ligation while His 254 is not involved in the Zn(II) coordination. However, His 254 plays a role in the stability of the OPH active site by stacking with His 230, which guides and stabilizes the Zn(II)-His 230 interaction.

### 2.3. The Double Mutant D253E-H254R

**Figure A3:**
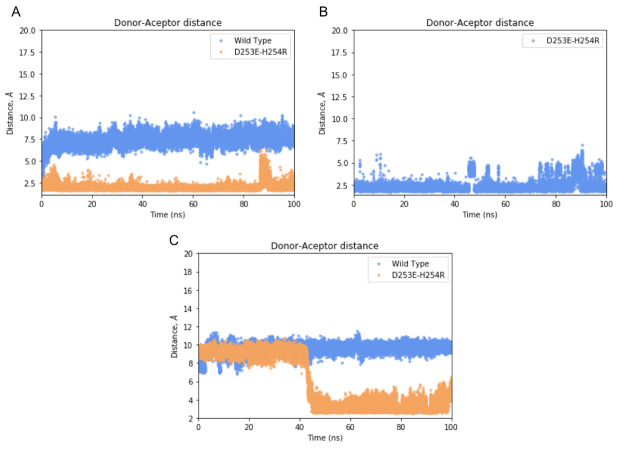
The donor-acceptor distances found during 100 ns of MD simulation on WT OPH complexed to paraoxon and the double mutant D253E-H254R complexed to paraoxon between: (**A**) The residue at position 254 (His in WT OPH and Arg in the double mutant) and Leu 271, (**B**) Arg 254 and His 257, and (**C**) Asp 100 and Lys 169. We found that during the simulations on the double mutant (D253E-H254R) complex, the distances between: (i) H/R 254 and Leu 271, (ii) Arg 254 and His 257, and (iii) Asp 100 and Lys 169 are short (fluctuating around 2 Å), signaling the formation of hydrogen bonds between the residues belonging to each of these pairs. The respective donor-acceptor distances during MD simulation on the WT OPH complex are greater than the typical distance of a hydrogen bond (2 Å). The distance between residues Arg 254 and His 257 corresponding to the WT complex are not shown on the plots of this figure since Arg 254 was found far from the residue at position 257 during the respective MD simulations. These results show that the double mutant D253E-H254R induced three different hydrogen bonds near the active site of OPH. Pictorial representations of these interactions are shown on Figure A4.

**Figure A4:**
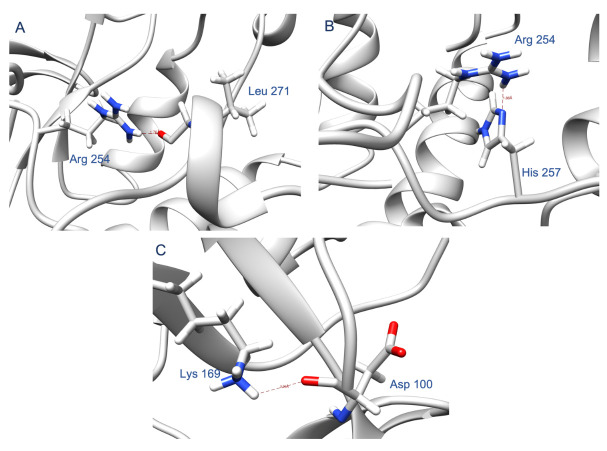
The donor-acceptor distances found during MD simulation on the double mutant D253E-H254R complexed to paraoxon between: (**A**) Arg 254 and Leu 271, (**B**) Arg 254 and His 257, and (**C**) Asp 100 and Lys 169. These results show that the double mutant D253E-H254R induced three different hydrogen bonds near the active site of OPH.

After identifying D253 as a hot-spot residue, we designed the double mutant D253E-H254R and tested its effect on interactions in the OPH active site using MD simulations. We chose to mutate position 254 since His 254 influences catalysis by interacting with active site residues, particularly His 230 (Figure 5A). Through comparing the donor-acceptor distances in the MD trajectory of the WT OPH to the donor-acceptor distances in the MD trajectory of the mutant form, we found that D253E-H254R induces three additional hydrogen bonds in the OPH active site. Out of these hydrogen bonds, two involve the side chain of Arg 254 Figure A3A,B and Figure A4A,B, which interacts with Leu 271, and His 257. The remaining hydrogen bond mediated by the double mutant occurs between Lys 169 and Asp 100 and appears after 40 ns of MD (Figure A3C and Figure A4C).

### 2.4. The Double Mutant D253E-I255G

**Figure A5:**
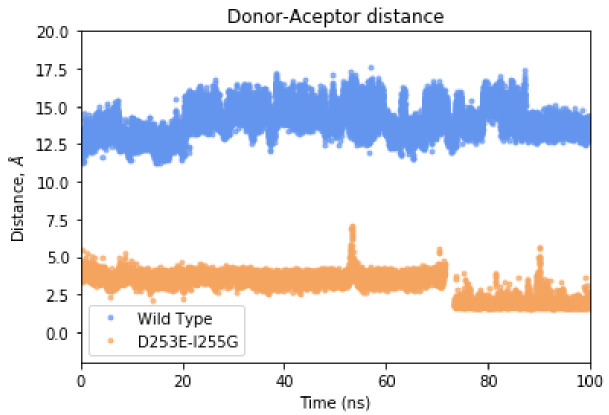
The donor-acceptor distances found during 100 ns of MD simulation on WT OPH:paraoxon complex and the double mutant D253E-I255G complexed to paraoxon between Asp 100 and His 257. These residues do not interact during the simulations of the WT complex. Asp 100 and His 257 are closer during the simulations of the double mutant complex compared to the simulations on the WT complex. In addition, these residues come close after 75 of MD on the mutant complex (D253E-I255G), where they establish a stable hydrogen bond with a distance fluctuating around 2 Å. This close contact is lost around 75 ns due to a wide opening of the protein’s structure which resulted in a distance between Asp 100 and His 257 higher than 20 Å, leading to missing data points around 75 ns. A pictorial representation of this interaction is shown on Figure A6A.

**Figure A6:**
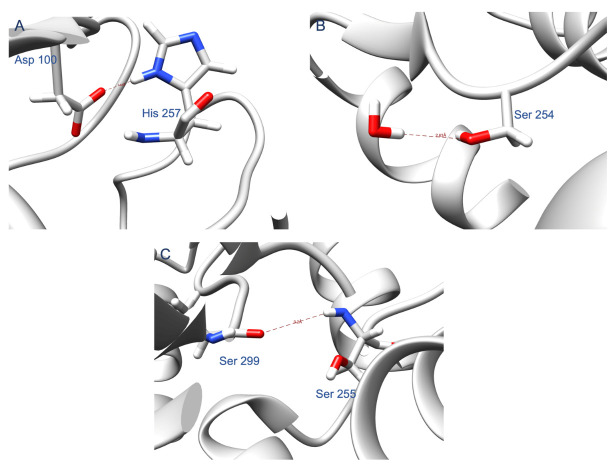
Hydrogen bonds formed during MD simulations on OPH mutants. (**A**) The hydrogen bond found during MD simulation on the double mutant D253E-I255G complexed to paraoxon between Asp 100 and His 257. (**B**) The hydrogen bond found during MD simulation on OPH:H254S between a crystal water molecule and the mutated residue H254S. (**C**) The hydrogen bond found during MD simulation on OPH:I255S between the mutated residue I255S and Ser 299.

The role of Ile 255 in the OPH active site is not described in the literature, to our knowledge. To test whether a subtle change near the important active site residues would affect the stability of the interactions and the flexibility of the substrate binding pocket, we coupled the mutation D253E with a second mutation at position 255. Then, we investigated whether the double mutation disrupted the coupled interactions observed in the OPH active site. Our MD simulations performed on the D253E-I255G mutant in complex with paraoxon showed the same hydrogen bonding profile observed during the simulations of WT OPH. One additional hydrogen bond between Asp 100 and His 257 was found in the trajectory of the D253E-I255G mutant form (Figure A5 and Figure A6A). This finding suggests that position 255 has an indirect role in stabilizing interactions in the OPH active site.

### 2.5. The Single Mutants H254S and I255S

Since the double mutants D253E-H254R and D253E-I255G induced changes in the interactions near the OPH active site and the single mutant D253E expanded the interaction network within the binding site, we decided to explore whether positions 254 and 255 are also important for the stability of the binding site. Thus, we designed two single mutants (H254S and I255S), where we changed the properties of the basic residue at position 254 and the non-polar residue at position 255, by replacing each of them with an apolar amino acid: Serine. Next, we performed MD simulations on the two complexes: OPH:H254S and OPH:I255S. We inspected the residues’ interactions in and near the binding site during the MD simulations and compared the hydrogen bonds found to those identified during the MD simulations on the WT complex.

**Figure A7:**
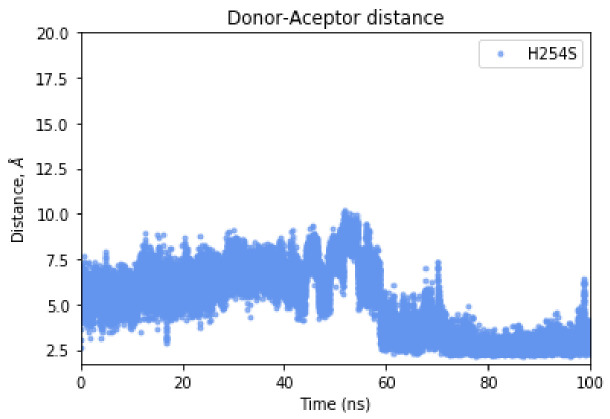
The donor-acceptor distance found during 100 ns of MD simulation on OPH:H254S between a crystal water molecule and the mutated residue H254S. After 60 ns of MD on the mutant complex (H254S), a water molecule belonging to the active site of OPH and involved in the coordination of Zn(II) establishes a hydrogen bond with Ser 254 with a distance fluctuating around 2.5 Å. A pictorial representation of this interactions is shown on Figure A6B.

For OPH:H254S, the same interactions were observed compared to WT:OPH. Only one hydrogen bond, detected between the mutated residue H254S and a water molecule in the binding site, appears after 60 ns of MD simulations on the mutant complex (Figure A7 and Figure A6B). It is worth noting that this water molecule is one of the two crystal water molecules that are involved in the coordination of one of the Zn(II) ions in the active site. This result suggests that the induced mutation, H254S, contributes to the stabilization of the Zn(II) coordination in the active site, and could perhaps improve paraoxon binding affinity.

**Figure A8:**
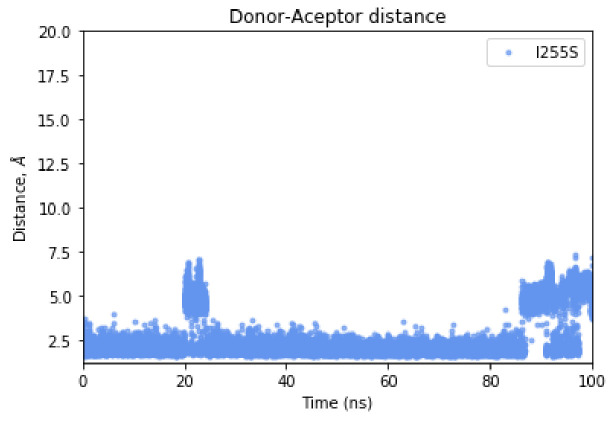
The donor-acceptor distance found during 100 ns of MD simulation on OPH:I255S between the mutated residue I255S and Ser 299. These two residues establish a stable hydrogen bond with a distance fluctuating around 2 Å. A pictorial representation of this interaction is shown on Figure A6C.

For OPH:I255S, the interactions established in and near the binding site are similar to those identified during the simulations of the WT complex. One stable hydrogen bond was induced by the mutation and formed between the mutated residue Ser 255 and Ser 299 (Figure A8 and Figure A6C). To our knowledge, S299 is not directly involved in the binding site activity, suggesting that the S255 mutation would not affect the stability of the active site and the catclytic activity.

### 2.6. Absolute Binding Free Energy Calculations on WT OPH and OPH Mutants

To obtain more detailed information about the affinity of paraoxon towards WT OPH and OPH mutant forms, we performed absolute binding free energy calculations in implicit solvent on these different complexes. Although binding free energy calculations in implicit solvent may not be as accurate as those in explicit solvent, we wanted to test the accuracy of this approach on our studied protein target and to assess the potential strength of OPH and OPH-mutants interactions with paraoxon within the limitations of the method.

**Figure A9:**
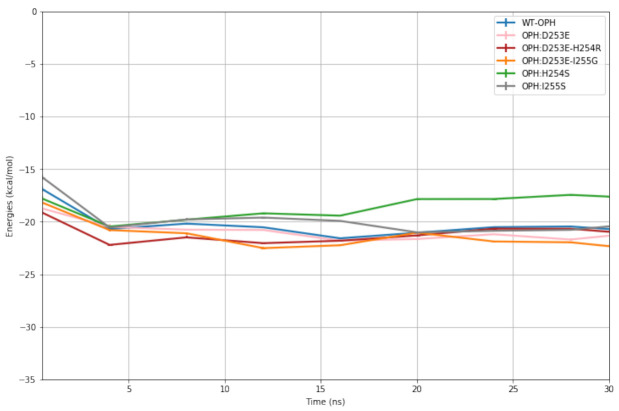
Absolute binding free energy calculations of OPH complexes. The binding free energies of paraoxon complexed with WT-OPH in blue, OPH:D2253E in pink, OPH:D253E-H254R in red, OPH:D253E-I255G in orange, OPH:H254S in green, and OPH:I255S in grey were computed in implicit solvent after 30 ns/thermodynamic state. Overall, the simulations are sufficiently converged and the binding of paraoxon to the mutant forms D253E, D253E-H254R, and D253E-I255G is more favorable than its binding to WT-OPH.

Overall, the binding free energies appear sufficiently converged with reasonable uncertainties (Figure A9 and Table 1). Three designed mutants show more favorable binding free energies to WT OPH with an improvement of 0.63 kcal/mol, 0.26 kcal/mol, and 1.63 kcal/mol for D253E, D253E-H254R, and D253E-I255G, respectively. These findings confirm that the single and the double mutations we introduced here enhance the stability of the substrate in the OPH active site.

Some of our other calculations, however, show that the single mutants (H254S and I255S) we designed to alter the property of the side chains at positions 254 and 255 failed to improve the binding of paraoxon to the protein. The calculated binding free energies of the mutants I255S (−20.44 kcal/mol) and H254S (−17.61 kcal/mol) are less favorable than that of the WT complex (−20.70 kcal/mol).

### 2.7. Spectrophotometric Assay of WT and Mutant OPH Activity

To evaluate the accuracy of our computational analysis, we experimentally tested the predicted mutations D253E, D253E-H254R, D253E-I255G, H254S, and I255S for catalysis of P-O bond cleavage of paraoxon. Using a continuous spectrophotometric assay, *k*_cat_ and *K*_M_ values were measured and compared to WT OPH (Table 2).

Notably, we found that the double mutant D253E-H254R exhibits a 19-fold increase in substrate binding affinity over WT. In addition, D253E and D253E-I255G enhanced the substrate binding affinity by 10- and 4.8-fold, respectively. In contrast, I255S did not improve the binding affinity of paraoxon significantly. These results exhibit a similar trend to the computational findings and suggest that hydrogen bonding within the OPH active site is important for stabilizing the OPH active site and enhancing substrate binding.

For H254S, our kinetic measurements show an improvement in substrate binding affinity by 5.2 fold compared to the WT form. This enhancement was not captured in our binding free energy predictions and might be due to the limitations of the computational method. One major limitation is the use of implicit solvent since the water structure could affect changes in protein-ligand complexes. In contrast, our MD simulations performed in explicit solvent align with the experimental results, supporting our speculations regarding the solvent. MD simulations show that H254S induces a hydrogen bond that stabilizes one of the Zn(II) coordinators, which could explain the substrate binding affinity of the enzyme. An additional limitation, of course, is that experimental measurement includes other factors, which is a function of more than just the binding free energy.

This study focused mainly on improving OPH substrate binding. While all mutants examined resulted in reduced values of *k*_cat_ compared to that of WT, the overall catalytic efficiency (*k*_cat_/*K*_M_) achieved for D253E, D253E-H254R and H254S mutants were still on the order of 10${}^{7}$ M^−1^ s^−1^, which approaches the limit of substrate diffusion (on the order of 10${}^{8}$ to 10${}^{9}$ M^−1^ s^−1^). Future work will address improvements to *k*_cat_ while maintaining the gains to *K*_M_ reported here.

## 3. Materials and Methods

### 3.1. Computational Methods

#### 3.1.1. Protein Preparation

For the computational part of this work, we used chain A of OPH from the PDB crystal structure 1HZY [14]. Chain A contains 330 amino acid residues and two Zn(II) in the active site. The first Zn(II) is coordinated by His 201, His 230, and two water molecules, while the second Zn(II) is coordinated by His 55, His 57, Asp 301, and a water molecule (Figure 2). In addition, we removed the carboxylic acid group originally added on the Lys 169 and treated residue 169 as a regular lysine. Based on the experimental conditions, we used the H++ server (http://biophysics.cs.vt.edu/H++ accessed on 7 December 2018) [15,16,17] to protonate the protein at pH 9. The resulting protein structure was used to prepare the different OPH mutants examined here—D253E, D253E-H254R, D253E-I255G, H254S, and I255S—using Chimera (University of California, San Francisco, CA, USA) [18]. Then, we used the MCPB.py program developed by Li and Merz [19] to determine the parameters of the OPH metal binding site. Merz-Kollman charges were obtained using Gaussian09 and the B3LYP/6-31G* level of theory.

#### 3.1.2. Docking

Starting from the SMILES string, we generated 1000 conformations of paraoxon using OMEGA 3.0.8 (OpenEye Scientific Software) [20]. Since most previous structural studies assume that the binding orientation of paraoxon is similar to that of the substrate analog [7,10,11], 4-methylbenzylphosphonate, we chose to perform the docking using three different tools from the OpenEye toolkits to evaluate whether these two compounds share a similar binding mode. These docking tools are: POSIT, HYBRID, and FRED (OEDocking 3.2.0; OpeneEye Inc.).

The three docking codes have different advantages. POSIT matches the 3D shape of a given reference ligand [21] and suggests a single docked pose that has a similar binding orientation as a reference ligand. HYBRID uses a reference crystallographic ligand to guide the docking process. Similar to POSIT, HYBRID uses a ligand-based scoring function that scores similar shape and 3D alignment between the docked ligand and the reference crystallographic structure. However, unlike POSIT, HYBRID uses the conformers of the ligand pre-generated with OMEGA and places them in several orientations in the protein active site. FRED does not use a reference ligand and outputs docked poses that complement the active site [22].

We used the docking codes for different purposes (Figure 3). The analog substrate structure, 4-methylbenzylphosphonate, was used as a reference ligand with POSIT and HYBRID. By using POSIT, we aimed to reproduce the binding orientation of 4-methylbenzylphosphonate and evaluate whether this is the correct binding mode of paraoxon, as described in previous studies. HYBRID was used to dock paraoxon with respect to the position of 4-methylbenzylphosphonate in the OPH binding site to suggest binding modes of paraoxon that are close to the binding mode of 4-methylbenzylphosphonate. Finally, FRED was used to test whether we can predict a stable binding mode of paraoxon independently of that of 4-methylbenzylphosphonate.

We generated fifty different docked poses using HYBRID with a resolution of 1 Å, then we visually inspected them using VIDA version 4.3.0 (OpenEye Inc.) to select the most dissimilar poses, with the goal of ensuring broad coverage of possible binding modes but allowing small variations in pose to be explored by subsequent simulations. We selected eleven different docked poses with respect to the position of the substrate analog in the binding site. Additionally, FRED was used to generate fifty different docked poses of paraoxon in the OPH binding site, with a docking resolution of 1 Å. Then, we performed root mean squared deviation (RMSD) clustering to choose the most dissimilar poses among the proposed fifty, within a cutoff of 2 Å. Consequently, two docked poses were selected in this step (Figure 3).

#### 3.1.3. MD Simulations

MD simulations on each selected docked pose in complex with OPH were carried out using the OpenMM simulation package 7.1.1 [23]. We assigned partial charges to the substrate (paraoxon) atoms using the AM1-BCC charge model [24]. We used tleap, a tool from the Amber16 package, to generate the water box and parameters. The protein and substrate were parameterized using Amberff14SB and GAFF version 1.8 force fields, respectively. The protein-substrate complex was then solvated using tleap with TIP3P water model in a cubic box with a 10 Å padding. Na^+^ and Cl^−^ counter ions were added to neutralize the system. We used the hydrogen mass repartitioning (HMR) approach [25,26] with a simulation time step of 4 fs. Long-range electrostatic interactions were calculated using the particle mesh Ewald method [27] with a cutoff of 10 Å for the real space electrostatics and Lennard–Jones forces. We minimized the water and ions for 4000 steps, while keeping the protein and the substrate restrained using 500 kcal/mol-Å^2^ positional restraints. Then, we performed a second minimization step on the water and ions for 4000 steps, with the protein and the substrate fixed using 50 kcal/mol-Å^2^ positional restraints. Next, we heated up the system from 10 K to 300 K in an NVT ensemble for 200 ps, while gradually releasing the restraints on the protein-substrate complex. In addition, we kept the positional restraints on the Zn(II) ions belonging to the active site of OPH during the minimization, equilibration and production steps. The temperature was controlled in the NVT simulations through Langevin dynamics with a collision frequency of 1 ps^−1^.

For the selected poses generated using POSIT (one pose), HYBRID (11 poses), and FRED (2 poses), the production run was done in an NPT ensemble for 15 ns, with the first 5 ns discarded as equilibration. Next, we evaluated the stability of the docking poses after MD and selected the most stable one. Specifically, we assessed the stability of the docking poses in the binding site by calculating the RMSD of each pose at the end of 10 ns of MD simulations with respect to the initial pose before MD (Figure 3). After this step, we extended the MD simulations of WT OPH complexed to the selected binding pose and ran additional MD simulations on the different OPH mutants in complex with the selected binding pose. For these simulations, the production run in the NPT ensemble was done for 105 ns, with the first 5 ns discarded as equilibration.

#### 3.1.4. Absolute Binding Free Energy Calculations

We performed binding free energy calculations using the alchemical approach, as implemented in YANK 0.17.0 (http://getyank.org/0.17.0), which is a framework for free energy calculations based on Hamiltonian replica exchange molecular dynamics simulations. After testing different protocols containing manually selected lambda values defining the thermodynamic cycle, we chose the ‘auto’ option provided by YANK to automatically generate an optimized set of lambda values for the WT OPH complex. Then, we used the same protocol to run binding free energy calculations on the mutant forms of OPH. For the complex phase, 43 lambda values were defined for the electrostatic and steric terms and the default value of 1.0 was used for lambda restraints in every thermodynamic state. For the solvent phase, 6 lambda values were specified for the electrostatic and steric contributions. We ran the simulations in an implicit/continuum solvent, namely Onufriev-Bashford-Case (OBC2) GBSA model [28]. All particle interactions were computed without any cutoff or periodic copes. We applied harmonic restraints [29] to keep the substrate near its binding site within the WT and mutant forms of OPH. These restraints were constant throughout the thermodynamic states.

The preparation of the OPH systems, which are WT OPH complexed to paraoxon and the five different mutants of OPH complexed each to paraoxon was done as described in the previous section: using AMBER ff14SB for the receptors and GAFF force fields for the substrate. Using YANK, each receptor was complexed with paraoxon and the respective receptor-substrate complex was solvated with OBC2-GBSA model. For the solvent phase, the files of the solvated paraoxon were generated through YANK. We ran 30 ns/lambda value for each of the described complexes.

### 3.2. Experimental Methods

#### 3.2.1. Cloning of WT OPH and Mutants into pET-24b(+)

The sequence containing the wild type (WT) *Pseudomonas diminuta* OPH gene was synthesized by GenScript (Piscataway, NJ, USA), and cloned into a pET24b(+) vector using NdeI/XhoI sites. The C-terminal stop codon was removed, and the His-tag sequence on the vector was used to express His-tagged OPH. Mutations of D253E, D253E-H254R, D253E-I255G, H254S and I255S in the pET24b(+) vector were also generated by GenScript (Piscataway, NJ, USA).

#### 3.2.2. WT and Mutant OPH Expression and Purification

The plasmid of WT or mutant OPH was transformed into the *E. coli* strain BL21. The cells were grown in 250 ml standard lysogeny broth (LB) at 37 ${}^{\circ }$C and optical density (OD) = 0.4, then induced by 1 mM Isopropyl β-D-1-thiogalactopyranoside (IPTG) at 16 ${}^{\circ }$C overnight. Cells were harvested by centrifugation at 4500×*g* for 15 min and resuspended in 5 ml of Ni-NTA lysis buffer: 50 mM NaH_2_PO_4_, 300 mM NaCl, 10 mM imidazole and 0.1 μM pepstatin at a pH of 7.4. The cells were lysed using Microfluidizer (Microfluidics, Westwood, MA, USA) and then centrifuged at 12,000× *g* for 30 min. The collected supernatant was incubated with 2 ml of Ni-NTA resin under end-to-end shaking and loaded on a 10 mL HisTrap FF column. After washing with buffer containing 50 mM NaH_2_PO_4_, 300 mM NaCl, 20 mM imidazole, the proteins were eluted with buffer containing 50 mM NaH_2_PO_4_, 300 mM NaCl, 250 mM imidazole.

#### 3.2.3. Determination of OPH Mutant Concentration

Series dilutions of purified recombinant OPH were used as standards. Expressed OPH mutants and standards were loaded onto an SDS gel, and OPH mutant concentrations were determined by densitometry using a Bio-Rad Gel Documentation system, version ChemiDoc (Bio-Rad Laboratories, Inc., Hercules, CA, USA).

#### 3.2.4. Spectrophotometric Assay of OPH Activity

Using a BioTek plate reader (Winooski, VT, USA), kinetic assays were performed by measuring the absorbance of the paraoxon leaving group at 405 nm over 10 min of reaction time. Various amounts of paraoxon (final concentration range: 0.001–3.0 mM) were added to 193.7 µL of buffer containing 100 mM CHES and 75 µM of Zn(OAc)_2_, pH = 9.0, reactions were started by adding 2 µL of WT or mutant OPH. The changes of absorbance at 405 nm over time were used to calculate initial velocities as a function of the various substrate concentrations. Then the initial velocity data, along with corresponding paraoxon concentrations, were plotted and analyzed by the Michaelis-Menten equation to obtain *V*_max_ and *K*_M_ using GraphPad (GraphPad Software, Inc., La Jolla, CA, USA [30]). The catalytic rate (*k*_cat_) was calculated by the ratio of maximum velocity (*V*_max_) and mutant concentration. Each kinetic measurement condition was performed in triplicate and standard deviation was calculated using GraphPad, version 6.0 (GraphPad Software, Inc., La Jolla, CA, USA).

## 4. Conclusions

Organophosphorus hydrolase is a bacterial enzyme that can detoxify a wide range of OP nerve agents. Despite thousands of engineered variants, OPH suffers from a low substrate binding affinity, hindering its action as an OP scavenger. Here, we focused on testing amino acid substitutions on residues near the enzyme active site to assess how these modulate substrate binding while minimizing the effect on enzyme catalytic efficiency. We computationally investigated single and double mutants to examine the effect of these mutations on the strong interactions occurring in and near the catalytic site. According to the modeling data, additional hydrogen bonds appear in the MD trajectories of the modified OPH enzymes. Hence, changes in the hydrogen bond network surrounding the active site may be important for the function of OPH and must be considered in the design of OPH variants. Strikingly, experimental measurements of kinetics showed that the predicted mutations improved substrate binding affinity compared to WT OPH substantially, thus confirming the computational predictions.

This work provides new insights guiding the protein design of organophosphorus hydrolase. Through coupling kinetic measurements and molecular modeling, we found hotspot residues that enhance the stability of paraoxon in the binding site and produced OPH mutants exhibiting better paraoxon binding properties. Two of the engineered mutants, D253E-H254R and D253E, enhanced the substrate binding of the enzyme significantly. Although both mutants resulted in reduced values of *k*_cat_, the overall catalytic efficiencies *k*_cat_/*K*_M_ achieved for both mutants were maintained on the order of 10${}^{7}$ M^–1^s^–1^. Additionally, we identified a stable binding mode of paraoxon that may help other researchers model OPH-substrate interactions. We expect that our structural findings highlighting the importance of the residues surrounding the active site of OPH and their strong interactions will assist future research. Combined with work on other important and extensively studied active site residues, these results could help further improve the catalytic function of OPH and its variants.

## Figures and Tables

**Table 1:** Absolute binding free energy calculations of WT OPH and the different OPH mutants. The binding free energies were computed in implicit solvent after 30 ns/thermodynamic state using a thermodynamic cycle of 43 states. Binding free energies (ΔG) and associated uncertainties were calculated using the Multistate Bennett Acceptance Ratio implemented in PyMBAR.

OPH Form	ΔG (kcal/mol)
WT	−20.70 ± 0.056
D253E	−21.33 ± 0.068
D253E-H254R	−20.96 ± 0.090
D253E-I255G	−22.33 ± 0.072
H254S	−17.61 ± 0.048
I255S	−20.44 ± 0.11

**Table 2:** Kinetic measurements of WT OPH and the different OPH mutants.

OPH Form	*k*_cat_ (s^−1^)	*K*_M_ (mM)	*K*_M_ Fold Improvement
WT	25,750.0 ± 2060.6	0.0835 ± 0.0186	-
D253E	128.0 ± 0.4	0.0083 ± 0.0012	10.0
D253E-H254R	346.4 ± 17.1	0.0044 ± 0.0010	19.0
D253E-I255G	46.5 ± 3.1	0.0172 ±0.0046	4.8
H254S	749.6 ± 41.9	0.0160 ± 0.0043	5.2
I255S	2.4 ± 0.1	0.0510 ± 0.0079	1.6

## Data Availability

The scripts and input files used for the computational part of this work are available free of charge on https://github.com/MobleyLab/OrganophosphateHydrolase.

## Abbreviations

The following abbreviations are used in this manuscript:

AChE	Acetylcholinesterase
DMBP	Diethyl4-methylbenzylphosphonate
MD	Molecular Dynamics
OP	Organophosphorus
OPH	Organophosphorus hydrolase
WT	Wild type
